# Patients’ perspectives on switching from one to three monthly Paliperidone Palmitate a cross-sectional patient satisfaction survey

**DOI:** 10.1192/j.eurpsy.2023.1046

**Published:** 2023-07-19

**Authors:** J. Barnett, S. Pappa

**Affiliations:** West London NHS Trust; 2 Faculty of Medicine, Imperial College London, London, United Kingdom

## Abstract

**Introduction:**

Paliperidone 3-monthly (PP3M) is a long-acting injectable antipsychotic (LAI) which has been shown to be an equally effective and more convenient alternative to Paliperidone 1-monthly (PP1M) (Hope *et al.* Australas Psychiatry 2018;26(2):206-209). A prerequisite for PP3M use is stability on a consistent dosing of PP1M ≥4 months, though, few studies have so far explored patients’ experiences with switching.

**Objectives:**

The aim of the study was to assess satisfaction and perspectives following the change to PP3M. A safety question with regards to the Covid-19 was also included.

**Methods:**

This cross-sectional survey was performed within a large, urban mental health setting between May-June 2021 while the UK was still under Covid-19 restrictions. Two psychiatrists obtained verbal consent before administering the survey. Questions 1 and 2 focused on satisfaction and safety with respondents rating to what extent they agreed or disagreed using a 5-point Likert scale. Questions 3 and 4 focused on advantages and disadvantages of the medication change; suggested answers were supplied but there was also an option to provide additional responses. Additional demographic and clinical information were collected from the electronic records.

**Results:**

Of the 61 patients who were receiving PP3M at the time of the survey 46 (31 male and 15 female) agreed to participate. One declined to participate, while 14 were not contactable, making the response rate 98% (46/47).

89.5% of respondents strongly agreed or agreed that they were satisfied after switching, 6.5% neither agreed nor disagreed and 4% disagreed. The bulk of the respondents (93.5%) strongly agreed or agreed that they felt safer having their injection every 3 months during the Covid-19 pandemic. 6.5% neither agreed nor disagreed but no one disagreed with this statement.

Questions on whether patients experienced any advantages or disadvantages as a result of the switch allowed for multiple answers. Convenience (93.5%), was the most popular positive reply, followed by improved quality of life (59%), decreased stigma (39%), better adherence (28%) and improved tolerability (21.7%). While 6.5% did not experience any advantages, 93.5% did not encounter any disadvantages, with 4.3% reporting worsening or new side effects and 2.2% a relapse of symptoms.

**Conclusions:**

The overall experience of switching to PP3M was positive. Similar to two previous studies (Pungor *et al.* BMC Psychiatry. 2021; 21, 300; Rise *et al.* Nord. J. Psychiatry 2021;75(4): 257-265) the majority of patients favoured the change quoting convenience, quality of life and reduced stigma as potential benefits. The importance of enhanced safety with less frequent medication administration under pandemic conditions was also highlighted. Shared and supported decision making should further inform clinical practice (Pappa *et al.* Community Ment Health J. 2021;57(8):1566–1578).

**Disclosure of Interest:**

None Declared

